# Habitat availability influences migration speed, refueling patterns and seasonal flyways of a fly-and-forage migrant

**DOI:** 10.1186/s40462-020-0190-4

**Published:** 2020-02-12

**Authors:** Thomas G. Hadjikyriakou, Emmanuel C. Nwankwo, Munir Z. Virani, Alexander N. G. Kirschel

**Affiliations:** 1 Department of Biological Sciences, University of Cyprus, PO Box 20537, 1678 Nicosia, Cyprus; 2 The Peregrine Fund, 5668 West Flying Hawk Lane, Boise, ID 83709 USA

**Keywords:** *Falco eleonorae*, Route selection, Fly-and-forage, GPS telemetry, Remote sensing, Africa

## Abstract

**Background:**

Despite our understanding of the principal factors that shape bird migration strategies, there is conflicting evidence regarding the role of habitat in shaping migration routes and schedules, including day and night activity and differences between autumn and spring. For fly-and-forage migrants, we predict that habitat characteristics might guide migration speed, route selection and migrating schedules.

**Methods:**

We use solar-powered GPS transmitters, obtaining high accuracy data, to monitor the migratory movements of Eleonora’s falcon breeding in Cyprus, which is the easternmost breeding population of the species. We tested for potential preferences in habitat characteristics along the migration routes, separately for the northern, drier part and the more vegetated southern part of the trips. We also examined the relationship between migration speed and vegetative cover during day and at night, accounting for wind support.

**Results:**

We found that tagged individuals repeatedly exhibited an anticlockwise loop migration pattern with spring routes being more easterly than autumn ones. We identified a preference for migration through vegetation-rich areas, where during daytime tagged individuals travel at slower migration speeds compared to vegetation-poor areas, indicating fly-and-forage activity. Birds roosted during most nights, combining refueling stopovers at selected vegetation-rich areas before or after crossing ecological barriers. Conversely, both during day and night, tagged individuals overflew unsuitable habitats more quickly.

**Conclusions:**

Our results suggest that habitat is an important factor in Eleonora’s falcon migratory strategies. Active selection of vegetation rich areas in combination with reduced migration speeds there, allows the migrating falcons to combine migration during the day with fly-and-forage refueling, while roosting most nights.

## Background

Migration is part of the annual cycle of many bird species that has evolved over millennia [1–3]. The main driving force is the exploitation of food resource fluctuations at certain times of year [4] avoiding resource depletion especially at breeding grounds [5]. Current bird migration patterns are thought to have evolved within the last 15,000 years, during postglacial species range expansions [2, 4, 6], resulting in complex movement systems [1, 3, 7, 8]. Despite their complexity, migration systems are characterized by high precision and accuracy, requiring high spatiotemporal understanding by the migrating birds [3, 9, 10]. Like any form of movement, migrations emerge through the interplay of internal and external factors [4], including genetic instruction, physiological processes and behavioral adaptations to external conditions such as ecological barriers [1, 2, 6, 7, 11, 12]. Yet, despite our understanding of the factors that generally dictate migration, less is known regarding how landscape characteristics, such as elevation, tree cover and habitat type, influence migratory movements [13]. Habitat might influence route selection and migration speed, resulting in differences in behavioural patterns between day and night, and autumn and spring [13]. In addition, individual repeatability between successive migratory trips might also be attributed to vegetation characteristics [14].

Avian migration strategies are guided by pre-migratory fuel deposition, but refueling en route is equally critical [15]. Integrating movement and environmental data can greatly enhance our understanding of how migrant birds balance travel with refueling opportunities [16–18]. An optimal strategy does not entail stopovers at all potentially fruitful refueling sites, which are instead selected according to fuel load, and perhaps using prior knowledge on food availability at specific areas over successive trips [15, 19, 20]. This combination can result in different migrating patterns, involving longer or shorter travel/refueling stints [21]. Furthermore, fly-and-forage activity, i.e. hunting while keeping to the general direction of travel, is specifically beneficial to aerial insectivores, which catch and consume prey on the wing [22]. Such species must therefore select flyways with insect-abundant habitats where they can keep on feeding while moving [23]. Ecological obstacles en route that do not offer feeding opportunities are either detoured or rapidly overflown [24]. While detours lengthen total distance travelled [25], they do not necessarily increase duration, as they could result from route optimization involving energy refueling and/or tailwind support [15, 26, 27]. Food availability and wind patterns are also thought to play a role in seasonal differences found in migration speed and trip duration [19, 28], as well as to observed loop patterns across autumn and spring migration [15, 29]. In addition, overall journey duration may be influenced by whether or not birds migrate at night [30]. Nocturnal migration typically provides more favorable climatic conditions, such as lighter winds and less turbulence, making flight more efficient [11, 22, 31]. For diurnally feeding species, nocturnal flight does not involve foraging activity [22], thus faster migration speed at night is expected [15]. As a strategy, nocturnal continuous flight with foraging during the day may result in shorter migration times [22]. Raptors, however, are not in general expected to fly at night [32] unless they are crossing large water bodies and need to extent their flight overnight [31, 33]. Nevertheless, high resolution tracking data might reveal more about nocturnal flying activity [34].

Eleonora’s falcon (*Falco eleonorae*) is a complete, long-distance, trans-equatorial migrant species [4, 11, 23], breeding primarily on islands and islets in the Aegean Sea, which is presumed to be its original range and the centre of its distribution [35]. The breeding population spreads from Cyprus in the east, westwards along the Mediterranean Sea, the Atlantic coast of Morocco and the Canary Islands [35]. Almost the entire population overwinters on the island of Madagascar [36], with a small proportion in the Mascarene Islands [35] and East Africa [37]. The species has evolved to take advantage of unique breeding and wintering niches in the Mediterranean and Madagascar respectively, utilizing abundant resources and avoiding interspecific competition [35]. Eleonora’s falcon feeds predominantly on insects most of the year, though during the breeding season it feeds primarily on migrating birds [38]. The delayed breeding period of Eleonora’s falcon coincides with the peak of autumn bird migration passage, with falcons catching migrant birds on the wing in order to feed themselves and their nestling during the offspring rearing period [35]. Previous telemetry studies on Eleonora’s falcon migration have shown that tagged individuals exhibited increased migration speeds over ecological barriers, compared to the slower migration speeds before and after crossing those barriers to refuel [39, 40]. Previous work also found that Eleonora’s falcons migrate during the day and at night throughout their trips [24]. In addition, an anti-clockwise migration pattern has been observed in Eleonora’s falcon between seasons, with spring routes being more easterly than autumn routes [40].

In this study, we utilized transmitters with GPS for accurate localization, providing also more consistent temporal data resolution compared to the transmitters previously used on Eleonora’s falcon migration studies [24, 39–41]. With those higher accuracy transmitters, we aimed to identify the extent to which migratory routes of Eleonora’s falcon target favored habitats with rich vegetation for stopovers and for refueling using a fly-and-forage strategy, adjusting their migration speeds accordingly. In order to examine specifically whether Eleonora’s falcon actively selects particular habitat characteristics, we compared use of those characteristics along the falcons’ migratory routes with the availability of those habitat features within their seasonal migration corridors. We examined if these habitat preferences could possibly vary between the northern (mostly desert) and southern (more vegetated) portions of their migration trips, as well as between seasons. In addition, if habitat influences flight and foraging decisions, we expect to find differences in migration speeds between habitats with different flying modes employed during the day and at night, such as soaring vs. flapping flight and fly-and-forage. If individuals move through different habitat types without changing their behaviour, especially their migration speed, this would suggest there is no habitat preference. Another possibility is that weather conditions may affect migration times, even resulting in stopovers when weather conditions are particularly harsh. We expected individuals to actively migrate at a faster migration speed, both during the day and at night over habitats unsuitable for foraging, such as extensive water bodies and deserts. We expect them though to migrate more slowly over insect-rich habitats and roost during the night therein. To that end we tested for the effect of vegetative cover and wind conditions on migration speeds, and examined how these effects could vary between the day and night and between seasons. In addition, obtaining data for more than one consecutive migration cycle for two individuals provided an insight into the spatial and temporal route repeatability of consecutive trips.

## Methods

### Field methods

Cyprus hosts around 130 pairs of Eleonora’s falcon nesting on the south coast of the island, from Cape Gata on the Akrotiri Peninsula in the east, to Cape Aspro in the west [42]. Between 2013 and 2017 we monitored the year-round movements of Eleonora’s falcons originating from Cyprus using for the first time GPS telemetry technology providing high spatial and temporal detail. In particular, we attached 12 transmitters on individuals breeding in the Akrotiri colony in Cyprus, of which eight, comprising five female adults, two male adults and one juvenile, provided us with migration related data (Table 1).

**Table 1:** Details of individuals, tag types, recorded trips and filtered locations

Tag type	Name of individual	Sex	Colour morph	Age	Date of capture	Autumn trips	Spring trips	Number of locations
GPS	Eleni	Female	Dark	4th year+	2/6/13	2	2	131
GPS	Ifigeneia	Female	Light	3rd year	7/6/13	1		334
GPS	Farofylakas	Female	Light	4th year+	10/9/13	4	3	658
GPS	Mitsis	N/A	Unknown	1st year	17/10/13	1		254
GPS	Pappous	Male	Light	5th year+	17/6/14	1		40
GPS	Forsman	Male	Light	4th year+	17/10/14	1		161
PTT	Aneti	Female	Light	4th year+	30/9/13	1		94
PTT	Tsampoukalou	Female	Light	4th year+	30/9/13	1		44
					TOTAL	12	5	1716

Birds were caught using mist nets at nesting and foraging locations [43]. Mist nets were placed on a cliff top opposite nests at the southern edge of the Akrotiri peninsula. A stuffed Eagle owl (*Bubo bubo*) was used as a decoy [44]. In addition, four nets were set within an olive grove, where the falcons had been observed feeding on large beetles at dusk. Birds were handled in accordance with standard procedures [45] and marked with metal rings [46], while biometric measurements including body mass were taken [47]. Sex and age was determined using colouration of bare parts and plumage characteristics [48]. Transmitters were attached with a teflon harness as a backpack [49]. Two different types of solar transmitters were used: ten GPS-GSM loggers (SKUA model; Ecotone Telemetry, Poland), and two Platform Transmitter Terminals (PTTs) (5 g solar PTT model; Microwave Telemetry Inc., Columbia USA). The weight of the transmitter ranged between 1.5 and 5% of the bird’s mass at capture [41, 50, 51]. No abnormal behavior was observed after transmitter attachment [52], with individuals moving as expected between breeding and foraging areas over subsequent days. GPS loggers collected locations on a user defined temporal pattern (2–6 h), and sent them through the GSM network, while PTT transmitters were set on a pre-programmed standard duty cycle of 10 h on followed by 48 h off [53].

### Data filtering

The positional error of GPS locations was less than 20 m for 80% of the retrieved points [54], we thus retained all available points for further analyses. The Kalman filtering algorithm was used to enhance the varying accuracy of PTT positions, which is divided in seven location classes (i.e., LC3 < 250 m, LC2 250–500 m, LC1 500–1500 m, LC0 > 1500 m, LCA and LCB = Unbounded accuracy, LCZ = Invalid location) [55]. Although LC3, LC2 and LC1 are typically considered as high quality (accuracy) locations [56], it was shown [57] that LCA had similar accuracy with LC1 while LC0 were far less accurate than LCA. Thus, in cases where we had to choose between which points to exclude (see subsequent filtering) we used the following order of priority: B, 0, A, 1, 2, 3. Consequently, points of all location classes apart from LCZ were used as long as they followed the general travel direction, assessed through visual inspection of the points in Google Earth [14, 44, 57, 58]. We excluded locations for both GPS and PTT transmitters that were obtained less than an hour apart to standardize sampling interval and for excessive autocorrelation avoidance [36, 59]. Furthermore, because of lower accuracy of PTT transmitters, we additionally excluded locations less than a kilometer apart [36, 60].

### Estimation of path metrics

Because of the lower accuracy of PTT obtained locations, we decided to rely only on GPS positions for path metric estimates using all available segments. Segment length between two successive telemetry fixes was calculated based on the geodesic distance [61], i.e. the shortest line between any two points on the earth’s surface considering the curvature of the earth [62]). In order to assign a position to day or night, sunset and sunrise information was obtained for each position based on local times [63], with half an hour before sunrise and after sunset included as daytime, because the species is also known to hunt at dawn and dusk [35].

Bird activity and flying mode between two successive telemetry fixes (e.g. resting, soaring, flapping and fly-and-forage) and related tortuosity of the tracks affect path metrics [64]. Therefore in this study, we define migration speed as the geodesic distance between the endpoints (successive telemetry fixes) of a segment, divided by the time between those points. Accordingly, calculated migration speed refers to the rate at which the migrant progresses along the geodesically defined path between two successive telemetry fixes. We considered active migratory movements those path segments where migration speed was >5 km/h [24, 31, 40], though such migration speeds might also include fly-and-forage activity. Because of potential overlap between fly-and-forage activity and forage-free migration speeds, we did not set a firm travel speed threshold to distinguish between these behavioural categories. Instead we simply considered that the higher the migration speed (accounting for wind support), the less likely the bird was foraging in flight.

### Habitat use at stopovers and roosts

As stopovers we considered areas with a 25 km radius where an individual stayed for at least 24 h without exhibiting directional migratory movement, allowing for one outlier point per stopover [44, 65]. This radius is sufficient to delineate stopovers as if birds exhibited directional movement towards the target destination, even with the lowest active migration speed of 5 km/h [24, 31, 40], flying only between sunrise and sunset (approximately 12 h), would cross this 25 km radius area during a 24 h period. Habitat use during night roosts, based on consecutive positions indicating that individuals were stationary, was identified by overlaying obtained migration positions onto a Moderate-resolution Imaging Spectroradiometer (MODIS) land cover layer [66]. Two resolutions of land cover were used; a finer available one with a resolution of 500 × 500 m (MCD12Q1) to reflect high GPS accuracy locations and a coarser one with about 5 × 5 km resolution (MCD12C1) to match the lower accuracy of PTT transmitters. To further utilize the high spatial accuracy of the GPS locations, Google Earth [67] was used to identify habitat details at roost and stopover areas, such as single trees within croplands, which could not be identified within remotely sensed habitat datasets [68].

### Route selection in relation to habitat characteristics

In order to determine whether Eleonora’s falcon selects specific habitat characteristics within its seasonal migration corridors, we compared actual bird positions with random locations generated within a 50 km buffer either side of each trip [69]. This distance is expected to be well within the range of vision of tracked individuals [70]. For each trip we therefore generated 150 random points in Geospatial Modelling Environment (GME) 7.2.1 [71] to provide a total number similar to the total number of recorded positions for use in statistical analyses. Because of the lack of specific information on food availability especially along tracks of long-distance migrants [72], we used the Normalized Difference Vegetation Index (NDVI) obtained from the MODIS sensor on the Terra satellite [66] as a proxy for the greenness along the migratory routes [68, 73] and as an indication of food availability for the migrating falcons [40, 74]. Sixteen-day NDVI granules (nominal resolution 250 × 250 m) were downloaded covering the extent of migration period and the granule that overlapped most with the duration of each trip event was used to extract the relationships separately. Data (actual and random) were also overlaid over a percent tree cover layer (hereafter ‘tree cover’) i.e. the vegetation continuous field product (nominal resolution 250 × 250 m) also from the MODIS sensor [66]. This more stable feature of the landscape, as well as elevation, have been found to influence movements and foraging activity of wintering falcons in Madagascar [75]. Elevation data were obtained from the United States Geological Survey website and had a resolution of 7.5 arc-sec, i.e. about 150 × 150 m [76]. All environment data were extracted using ArcGIS 10.1 [62]. For all relationships of locations with environmental data (NDVI, percent tree cover and elevation), we extracted bilinear interpolated data, which incorporate adjacent cell values in the value calculation, to allow for a uniform approach for both the higher resolution GPS data and the lower resolution PTT obtained data. For habitat selection analyses we excluded actual locations and random points over the sea and other extensive water bodies for which NDVI and tree cover were not available. We investigated which environmental characteristics affect route selection of Eleonora’s falcon during migration with the use of Generalized Linear Mixed Models (GLMM) with a binomial distribution and logit link function in lmer in R 3.5.1 [77]. Specifically, we tested for the effects of NDVI, tree cover and elevation and their interactions with season as fixed effects, individual and year as crossed random factors, with a binary value for whether a point represented an actual position or a random point as the dependent variable. We ran these models separately for the northern, drier (e.g. Sahara desert), and southern (e.g. savannah and rain forest) parts of the migration journeys. To set latitude separating these north and south sections of the trips, we used the MODIS land cover layer [66] as an indicator to identify the desert and non-desert areas, setting the line at latitude 16^o^N. We used the Corrected Akaike’s Information Criterion (AICc) to select the best models for each test, considering all models with ΔAICc < 4 compared to the best supported model.

### Migration speed differences in relation to season, habitat characteristics and daily activity

Because migration speed is related to the activity of tracked individuals, for example slower migration speeds are expected when birds are foraging, we investigated which factors affect the migration speed of Eleonora’s falcon during migration with the use of GLMMs with a Gaussian distribution with identity link function using lme4 in R 3.5.1 [77]. Specifically, we tested for the effects on migration speed of tailwind, NDVI, percent tree cover (all centred and standardized, [78]) and season, as well as the interactions between the environmental variables and season. Individual and year were included as crossed random factors to account for spatial and temporal autocorrelation. Because of the expected differences in flight modes between day and night, with night flights not affected by thermals, and expectations of reduced foraging at night, we ran separate models without the transitioning segments between day and night. Specifically, we investigated how NDVI, tree cover, tailwind and season affect migration speed during the day and at night, including the interactions of each factor with day and night. We ran a separate model including all recorded flights using the dataset excluding transitioning segments, and another including only flights over 5 km/h to focus specifically on active migratory and fly-and-forage migration speeds, when landscape characteristics can influence movements differently compared to stationery periods, especially in relation to food availability. Because of the smaller sample size of segments over 5 km/h, we ran Bayesian GLMMs in blme. We used AICc to select the best models for each test, while also considering all models with ΔAICc < 4 compared to the best supported model.

### Accounting for confounding wind effects

Knowing that wind will have an effect on migration speeds, and to make sure that the wind effect is accounted for when estimating the independent effect of habitat characteristics, we included wind as a fixed effect in our models. The loggers that we used did not provide flying altitude data, so to calculate tailwind at each GPS location we used winds at 850 hPa pressure level, corresponding to a mean altitude of 1500 m a.s.l.. In previous studies on raptors migrating through Africa a 925 hPa pressure level (mean altitude of 750 m a.s.l.) has been used, but most of those studies were conducted on the western African flyway (e.g. [29, 68, 79]), where ground elevations are considerably lower than for the eastern flyway studied here. Indeed, mean ground elevation of telemetry locations in the present study was 750 m a.s.l.. Data downloaded from ERA5, the latest atmospheric reanalysis product of the European Centre for Medium-Range Weather Forecasts (ECMWF) [80]. U (east-west) and V (north-south) components for each telemetry location were downloaded in R using the NCEP.interp function in RNCEP [81]. These two wind components were combined to provide tailwind speed in relation to the overall migration direction [65] of 170^o^ degrees for autumn and 350^o^ in spring, based on the shortest direct line potential migratory route from Akrotiri colony in Cyprus to the point of the narrowest crossing over the Mozambique Channel to Madagascar and vice versa.

## Results

### Description of migratory routes

From the eight Eleonora’s falcons tagged that provided migration data (Table 1), we obtained data for seven migration seasons (four in autumn and three in spring) between 2013 and 2016, from a total of 17 individual migratory events (12 autumn and five spring). Following data filtering of low-quality positions and eliminating potential sources of autocorrelation, we retained 1716 positions (1578 GPS and 138 PTT).

**Fig. 1:**
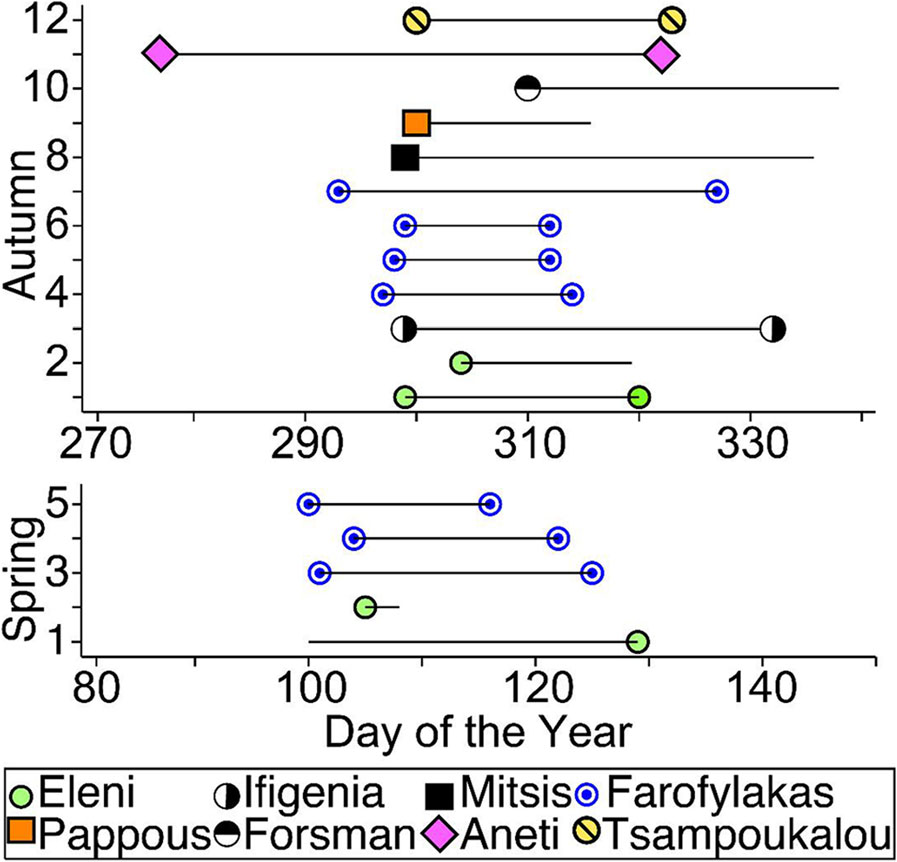
Recorded departure and arrival dates for Eleonora’s falcon migration events during autumn and spring. Lines indicate duration between departure and arrival (Madagascar and Cyprus) while symbols represent exact departure and arrival dates for each trip where known (each individual is represented with different symbol as shown in the legend, with consecutive trips for repeatedly tracked individuals bunched together). To aid comparison, day of the year was used. The peak onset of southbound migration was between 25 – 27th October, and northbound between 9 – 15th April

Autumn migration duration was on average longer (*N* = 8, mean = 24.75 days, SD = 11.52) than spring migration (*N* = 3, mean = 19.33 days, SD = 4.16), though we note that the confirmed durations in spring came from only one individual (Farofylakas) for which the comparative results indicate the same average duration (autumn: *N* = 4, mean = 19 days, SD = 10.03; spring: *N* = 3, mean = 19.33 days, SD = 4.16). In autumn, departure from Cyprus occurred between 4th October and 6th November, with the peak around 26th October (eight out of 12 confirmed departure dates falling between 24th and 27th October). Arrival in Madagascar occurred between 8 and 28th November without any obvious peak. In spring, departure from Madagascar was between 9 and 15th April, and arrival in Cyprus between 25th April and 9th May (Fig. 1).

**Fig. 2:**
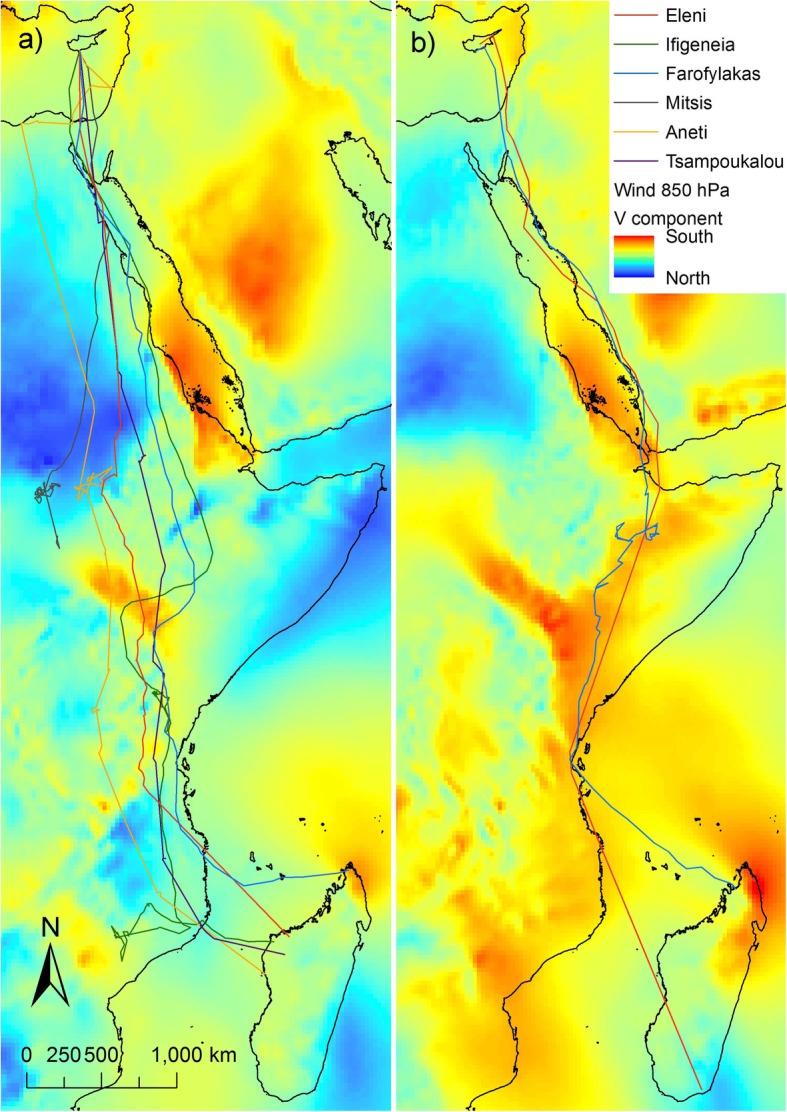
Migratory routes during **a** autumn 2013 and **b** spring 2014 overlaid on to the respective monthly north – south winds (V wind component) at 850 hPa (c1500 m a.s.l.). Line colors represent the different individuals. Autumn routes through the Sahara desert were aided by prevailing northerly winds, while spring routes east of the Red Sea, avoid Sahara predominant headwinds. Data downloaded from ERA5 atmospheric reanalysis product of the European Centre for Medium-Range Weather Forecasts (ECMWF) [80]

**Fig. 3:**
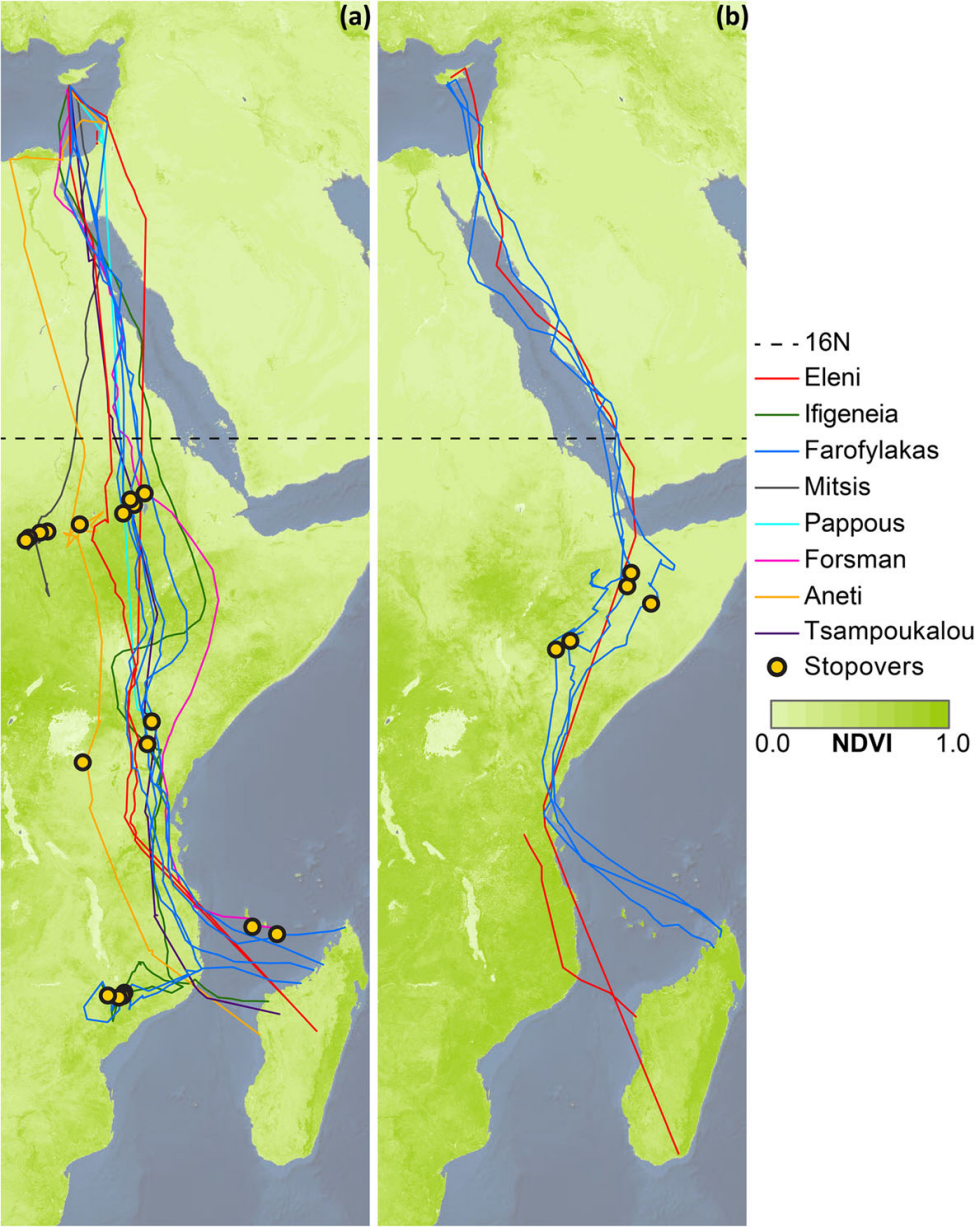
Migratory routes during **a** autumn and **b** spring overlaid on to the respective NDVI raster data for each season [66]. Line colors represent the different individuals, while yellow points denote stopovers. Autumn routes were western than spring ones resulting in a loop migration pattern. Dotted line denotes 16^o^N latitude which was used to separate locations used for actual positions vs. random points analysis in northern (drier) and southern (more vegetated) portions of the migratory routes

**Fig. 4:**
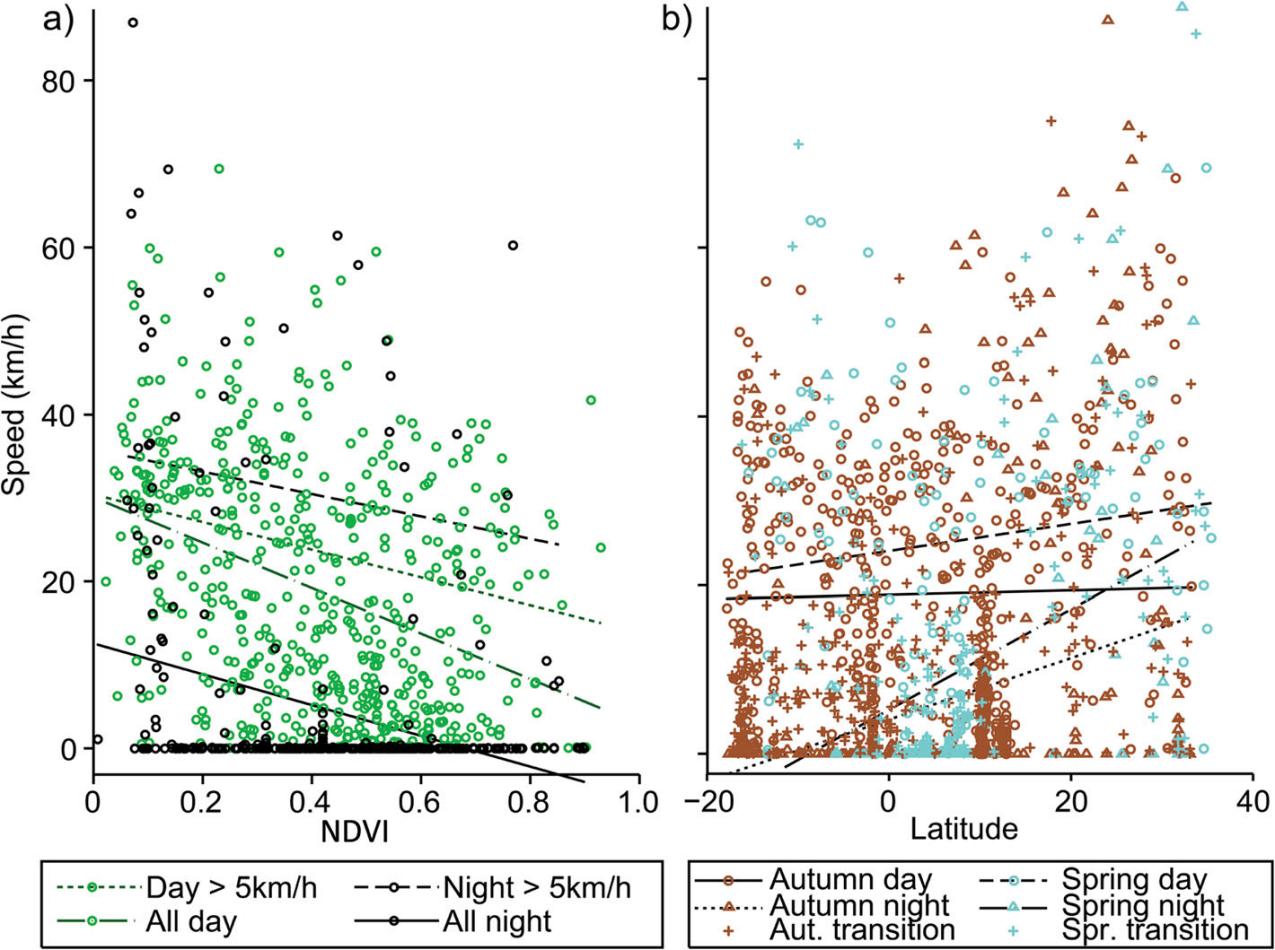
**a** Migration speeds over NDVI with lines of best fit, during day and night, for all segments, and for those segments described as active migration (migration speeds > 5 km/h), but excluding transitions between day and night. Migration speeds were significantly slower over areas with higher NDVI, with slower migration speeds overall recorded at night suggesting birds roost during most nights. By including only presumed active migratory movements (migration speeds > 5 km/h), migration speeds were typically higher at night than during the day. **b** Migration speed over latitude for autumn and spring, including night, day and transition flights between day and night. There are sites at specific latitudes with concentrations of points, which indicate stopovers for roosting and foraging

**Fig. 5:**
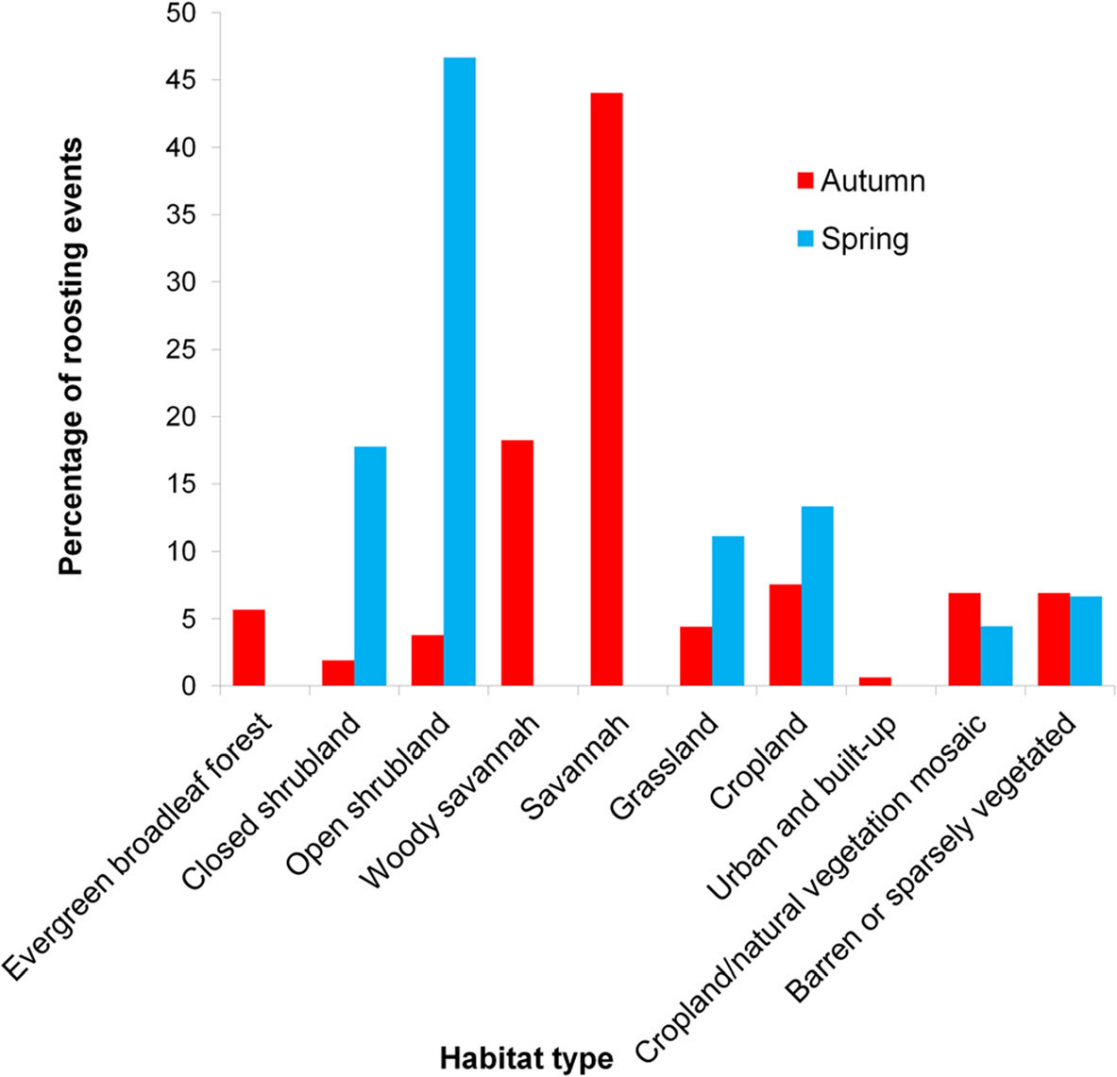
Roosting habitat types in autumn and spring. The majority of roosts in autumn were in savannah (62%), while in spring they were in shrublands (65%)

In autumn, upon initiating migration, tagged falcons appeared to fly non-stop south. Tagged individuals crossed the Mediterranean from Cyprus and from there most routes were over the Sinai Peninsula along the western coast of the Red Sea and over the Sahara Desert at its narrowest crossing in the east, aided by predominant northerly winds (See Fig. 2a for north-south winds and Additional file 1: Figure S1a for east-west winds) [3, 80]. After crossing the Sahara, individuals roosted for most nights from there onwards, including one or two stopovers for most routes within savannah in Ethiopia, Sudan or Kenya. All routes continued southwards towards Mozambique, from where on occasion individuals headed westwards for refuelling stopovers prior to the crossing of the Mozambique Channel to Madagascar (Fig. 3a). In spring, the tagged falcons crossed the Mozambique Channel, at times straight over Zanzibar to Tanzania. Then, aided also by predominant winds (See Fig. 2b for north-south winds and Additional file 1: Figure S1a for east-west winds), moved through eastern Kenya to Ethiopia and Somalia where tagged individuals had a short stopover before following a more easterly route than in autumn, primarily along the east coast of the Red Sea, avoiding Sahara predominant headwinds (Fig. 2b) [3, 80], and then across the Mediterranean to the breeding grounds in Cyprus (Fig. 3b). The migration speed over latitude relationship shows that there were sites at specific latitudes with concentrations of stopovers for roosting and foraging. The autumn concentration of points around 10–13° N, suggests that there was a major stopover upon reaching savannah after rapidly overflying the Sahara desert. After this refuelling stop there were further autumn concentrations representing stopovers, especially around −2° S and − 15° to − 17° S. The main stopover in spring appeared to occur around 8° to 9° N. In addition, very few night roosts were observed from 13° to 32° N, indicating continuous flight day and night (Fig. 4b). In autumn, tagged individuals roosted predominantly in savannah (62% of roosting nights), while in spring they roosted mostly in shrubland (65% of roosting nights) (Fig. 5).

### Route selection and migration speed in relation to habitat characteristics

The average distance covered in autumn was 7139 km (SD = 1320), compared to 7245 km (SD = 401) in spring. The shortest path distance was 5749 km (SD = 209) in autumn and 5845 (SD = 626) in spring, so actual distances traveled were about 24% further than the minimum possible distance. The longest apparently continuous flight, recorded for Ifigeneia, was 3530 km, covered in 85 h with an average migration speed of 40 km/h (maximum single segment migration speed 73 km/h). We found that during the crossing of the drier (northern) part of their migration routes, the falcons used areas with higher NDVI in lower elevations compared to generated random points within the 50 km buffer zone either side of their path (Table 2a). In the more vegetated (southern) part of their migration routes, tagged individuals did not specifically move over areas with higher NDVI, though they did select areas with denser tree cover, especially in spring, and at higher elevations particularly in autumn, compared to what was available to them along their migratory paths (Table 2b).

**Table 2:** Significant results of binomial GLMMs from the best supported models showing in which ecological factors actual positions differ from random points generated within a 50 km buffer zone either side of the flying path. Specifically, the effects of NDVI, tree cover and elevation and their interactions with season were tested for the a) northern (latitude 16 N northwards) and b) southern (latitude 16 N southwards) parts of the trip. In the drier north (e.g. Sahara desert), the falcons used areas with higher NDVI compared to randomly generated points, avoiding higher elevations. In the more vegetated south, the preference for higher NDVI was not significant, tagged individuals occurred though (especially in spring), in areas with higher tree cover, and subsequently higher elevations (particularly in autumn) than what was available to them along their route corridors. Best supported models were selected based on lowest AICc values (Full list of candidate models and results from full model and models within ΔAICc < 4 in Additional file 1: Table S1 and S2)

Variable	Estimate	SE	z	P
a) North (*N *= 901)				
Intercept	9.23	1.35	6.84	< 0.001
NDVI (log)	−3.07	0.39	−7.80	< 0.001
Elevation (log)	0.52	0.17	3.04	< 0.01
b) South (*N *= 2811)				
Intercept	3.50	1.03	3.42	< 0.001
Tree cover (log)	−0.48	0.10	−4.85	< 0.001
Elevation (log)	−0.34	0.12	−2.92	< 0.01
Tree cover (log): Season	0.49	0.17	2.94	< 0.01
Elevation (log): Season	−0.17	0.06	−2.88	< 0.01

**Fig. 6:**
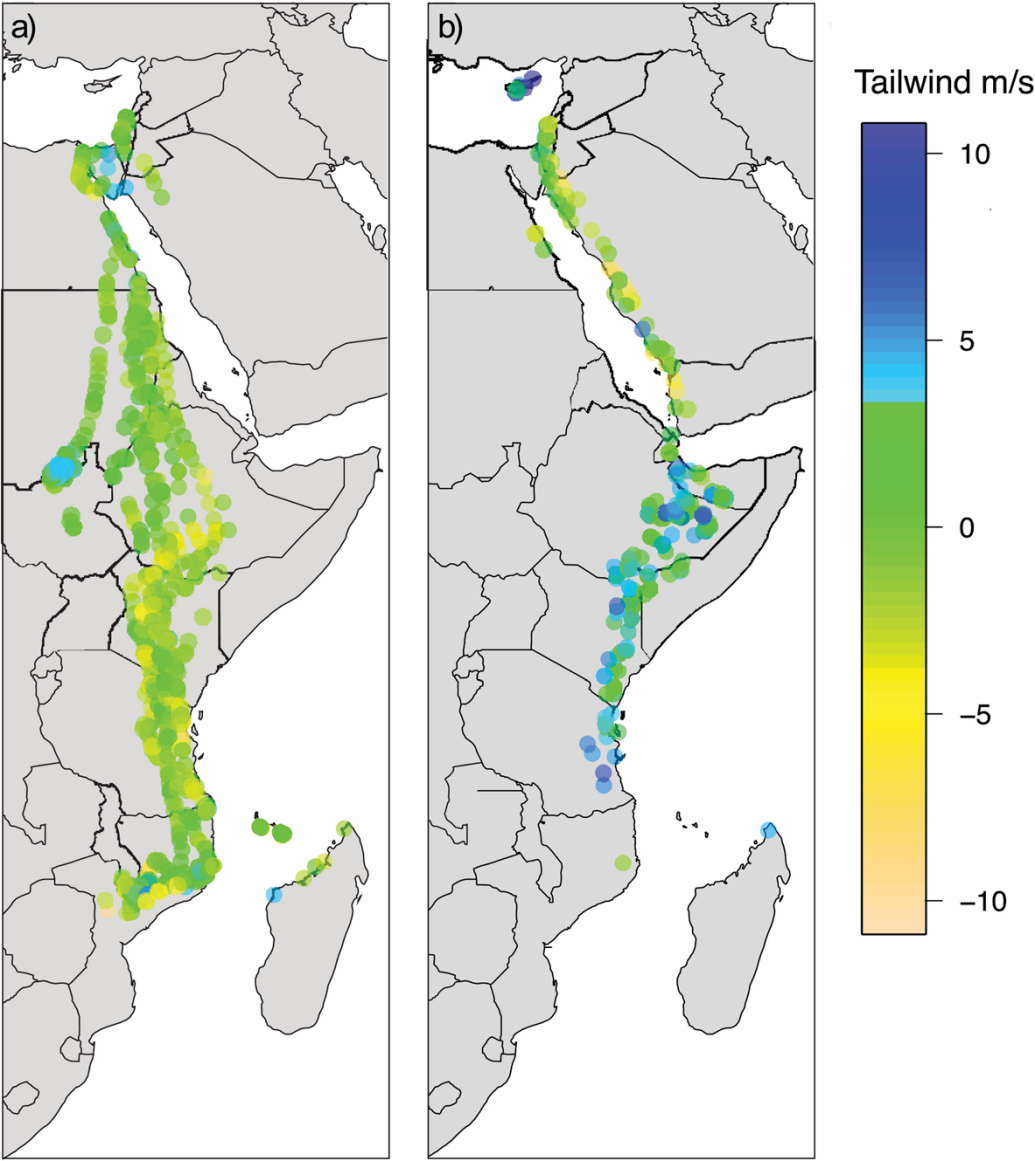
Tailwind support for all telemetry locations for **a** autumn and **b** spring migratory routes. Tailwinds appear stronger during the northern part of the journeys in autumn, predominantly over the Sahara desert. On the contrary, tailwinds appear stronger on the southern part of the spring migration routes

Birds mostly roosted at night, with an average diurnal and nocturnal migration speed of 20.02 km/h (*N* = 588, SD = 15.33) and 6.41 km/h (*N* = 474, SD = 15.94) respectively. The highest migration speeds though were recorded at night, both in autumn and spring, over the Mediterranean, Sinai and the Red Sea with a maximum migration speed of 89 km/h recorded. Overall, migration speeds were significantly slower over areas with higher NDVI and higher tree cover and also significantly slower in spring compared to autumn because of the relative effect of tailwinds (Table 3a). After excluding the transitioning segments between day and night, slower migration speeds were recorded at night and over areas with higher NDVI (Fig. 4a), with differences in migration speed between day and night being greater in autumn than in spring (Table 3b). After filtering out those segments with migration speeds < 5 km/h, we found that migration speeds were higher with increasing tailwind (Fig. 6). Migration speeds were still slower over higher NDVI, but conversely, higher during the night (*N* = 84, mean 35.61 km/h, SD = 19.98) compared with the day (*N* = 454, mean 25.42 km/h. SD = 13.25; Fig. 4a). There was a seasonal effect in this active migratory movement dataset, with the difference in migration speed between day and night being again higher in autumn compared to spring, while there was a negative interaction effect of day and night and tree cover, indicating that falcons travelled more quickly at night compared to the day over less vegetated areas (Table 3c).

**Table 3:** Significant results of GLMMs from the best supported models showing the effects of NDVI, tree cover, tailwind (all centered and standardized [78]) and season on migration speeds, with individual and year as crossed random factors for a) all GPS telemetry based segments, b) excluding transitioning segments between day and night and including day vs. night as a fixed factor along with its interactions with the other predictors, and c) comparing day and night again, but this time focusing just on active movements (migration speeds >5 km/h). (Full list of candidate models and results from full models and models within ΔAICc <4 in Additional file 1, Table S3, S4, S5)

	Estimate	SE	t	P
a) All GPS telemetry locations (*N* = 1435)				
Intercept	−0.02	0.11	−0.19	0.86
NDVI	−0.34	0.03	−13.52	< 0.001
Tree cover	−0.08	0.03	−2.52	< 0.05
Season	−0.22	0.08	−2.87	< 0.01
Tailwind: season	−0.22	0.06	−3.45	< 0.001
b) GPS telemetry locations excluding transitions between day and night (*N* = 984)				
Intercept	0.46	0.13	3.65	< 0.05
Day/night	−0.89	0.06	−14.67	< 0.001
NDVI	−0.33	0.03	−11.10	< 0.001
Season: day/night	−0.44	0.13	−3.34	< 0.001
c) Active movements only (*N* = 465)				
Intercept	−0.19	0.20	−0.94	0.35
Day/night	0.70	0.15	4.81	< 0.001
Tailwind	0.10	0.05	2.06	< 0.05
NDVI	−0.26	0.04	−5.97	< 0.001
Tree cover: day/night	−0.30	0.10	−2.85	< 0.01
Season: day/night	−1.13	0.30	−3.73	< 0.001

## Discussion

In spite of the observed loop migration pattern, both average distances covered and migration duration were found to be similar between seasons, in contrast with findings for birds tagged in the western Mediterranean [40], though our results are based on a small sample size. On the other hand, departure dates from breeding grounds are in agreement with results from other colonies [24, 39, 41], while in spring the narrow migration window we found also coincides with that found for birds breeding in the western Mediterranean [40].

Despite the small sample size, we believe that the relatively small latitudinal temporal differences between consecutive trips and the relatively narrow migration corridors between consecutive routes, suggest some individual repeatability. This contradicts results for birds tagged in the western Mediterranean for which no individual repeatability was identified, with larger longitudinal distances between consecutive trips [40]. Eleonora’s falcon overwinters in a comparatively small area, primarily in Madagascar, indicating that in addition to internal mechanisms of orientation [24], the species also uses external navigation cues [41]. As soon as migrants establish an optimal route and survive, they remember and copy it for consecutive years [6], enhancing migration success [23].

The preference for vegetation-rich areas with active selection of higher NDVI over drier areas in the north and higher tree cover further south, in conjunction with the observed slower migration speeds over those vegetation-rich areas, indicate that birds spend time refueling in vegetation-rich areas, possibly with fly-and-forage strategy, thus reducing the necessity for regular stopovers [13]. In addition, because of the high spatial accuracy and more consistent temporal position collection of this study with the use of GPS transmitters, we were able to show that in contrast to findings for birds breeding in western Mediterranean [24], birds roosted during most nights in vegetation-rich areas primarily in savannah in autumn and shrublands in spring, resulting in significantly slower migration speed at night. Despite that overall trend, when considering only active movements (migration speed > 5 km/h), migration speeds were significantly faster at night, when the birds would not be foraging when flying, with possibly better flying conditions as well [22], further supporting the daily fly-and-forage assumption. Even when we account for migration speed differences due to variation in wind support, we found that falcons travel at significantly higher migration speeds over vegetation-poor areas, day and night. This is in line with recent findings that upon facing the challenge of crossing an ecological barrier, birds are able to adapt their diurnal and nocturnal flight patterns to overcome it as quickly as possible [82]. This is in accordance with expectations [22] and supports the hypothesis that vegetation-poor areas are ecological barriers for Eleonora’s falcon [24], which they overcome by rapidly traversing rather than seeking a detour [24, 39, 40], with higher migration speeds over sea and desert [83]. This indicates that landscape characteristics are guiding the routes and the migration speed [13], with individuals behaving as ‘sprinting migrants’ when feeding opportunities are lacking, such as over the Sahara desert [13]. Characteristically, the remarkable non-stop flight by one individual for 3530 km in 85 h, a first such record for Eleonora’s falcon and one of the longest trips recorded for any species [3], indicates the capacity of Eleonora’s falcon to travel extensive distances, over sea and other ecological barriers, such as deserts, without refueling. Lesser kestrels and Eurasian hobbies have been recorded flying up to 1626 km and 740 km respectively, probably non-stop, during migration [44, 84], but Eleonora’s falcon appears to be capable of much longer flights.

In autumn, the Sahel region south of the Sahara is quite green with an abundance of flood pools and insects [3], and birds benefit from refueling there after the Sahara crossing. On the contrary, in spring the Sahel is at its driest, with potentially less food available [3], possibly contributing to the observed loop pattern. The short rainy season in eastern Ethiopia in spring might facilitate movements through there because of a timely abundance of potential insect prey [3], as suggested similarly for Red-backed shrikes [83]. This agrees with field records during spring migration, where Eleonora’s falcons have been observed hawking on large insects and perching on trees in Somalia following the rains [35]. The occasional delay in crossing the Mozambique Channel in autumn could be attributed to unfavorable weather conditions, or alternatively an expected need to refuel before crossing over an ecological barrier [85], as found in other studies [39, 41]. The suggestion though that this stopover area towards Malawi can be considered as part of the wintering grounds of Eleonora’s falcon [36, 41] is supported by our results here and deserves further investigation.

We suggest that the loop migration pattern is most likely attributed to habitat availability and the need for refueling, in agreement with previous studies [39, 40], yet, although long distance migrants are constraint by distance and time, and are thus expected to be less selective to wind conditions compared to short distance migrants [33], the effect of wind cannot be overlooked. Northerly tailwinds appear to aid the southbound Sahara crossing [3, 80]. On the other hand in spring, prevailing winds might contribute to the eastwards shift towards Ethiopia and Somalia [80]. From there tagged individuals cross the Red Sea at its narrowest point at Djibouti and remain east of the Red Sea, avoiding stronger headwinds west of the Red Sea (Fig. 2) [3, 80, 83]. Interestingly, this anticlockwise pattern in Eleonora’s falcon is contrary to the overall trend in many species, where autumn migratory routes are more eastern than spring ones in the Palearctic [86]. Indeed, a clockwise migratory loop pattern has been found in Lesser kestrel [44], Marsh harrier [87], and Egyptian vulture [88], but this was attributed to dominant wind patterns between the two migration seasons in West Africa where wind patterns differ from our study area.

## Conclusions

Multi-year monitoring provides us with a greater understanding of migration cycles [14] and our 4-year study is the longest telemetry study on Eleonora’s falcon thus far. Apart from duration, this study is the first to use GPS-GSM loggers on Eleonora’s falcon, providing opportunities for more detailed information on migratory routes [89], and allowing for more accurate explanations of bird migratory strategy in relation to wind, vegetative cover and feeding opportunities [13, 90]. More long-term migration studies with high accuracy transmitters providing flying altitude, will further contribute to our understanding of migratory strategies that in turn can better inform conservation efforts for long distance migratory birds during what is often the least well studied portion of their annual cycle [13, 91].

## Supplementary information

**Additional file 1: Table S1.** GLMM results for comparison between actual positions and random points generated within a 50 km buffer zone either side of the flying path, in relation to NDVI, tree cover and elevation for the north part of the migratory routes (latitude 16 N northwards) (*N* = 901): a) full list of candidate models; b) full model; c) candidate model (ΔAICc <4); d) candidate model (ΔAICc <4); e) candidate model (ΔAICc <4); f) candidate model (ΔAICc <4); g) best supported model. In the drier north (e.g. Sahara desert), the falcons used areas with higher NDVI compared to randomly generated points, avoiding higher elevations. **Table S2**. GLMM results for comparison between actual positions and random points generated within a 50 km buffer zone either side of the flying path, in relation to NDVI, tree cover and elevation for the south part of the migratory routes (latitude 16 N southwards) (*N* = 2811): a) full list of candidate models; b) full model, c) candidate model (Δ AICc < 4); d) best supported model. In the more vegetated south they occurred (especially in spring), in areas with higher tree cover, and subsequently higher elevations (particularly in autumn) than what was available to them alongside their routes. **Table S3**. GLMM results for the effect of all GPS telemetry locations (*N* = 1435) on migration speed, of NDVI, tailwind, tree cover and season, with interactions of season with the other fixed effects, and individual and year as crossed random factors: a) full list of candidate models; b) full model; c) Candidate model (ΔAICc < 4); d) Candidate model (ΔAICc < 4); e) Candidate model (ΔAICc < 4); f) best supported model. Migration speeds were significantly slower over areas with higher NDVI and tree cover and also significantly slower in spring compared to autumn because of wind effect. **Table S4**. GLMM results for the effect on migration speed, of NDVI, tailwind, tree cover, season and day/night (excluding transitioning segments between day and night) with the interactions of day/night with the other fixed effects and individual and year as crossed random factors (*N* = 984): a) full model; b) best supported model (Delta AICc < 4). In this dataset, slower migration speeds were recorded at night, and over areas with higher NDVI. Differences in migration speed between day and night were greater in autumn compared with spring, with significantly slower migration speeds at night during spring. **Table S5.** GLMM results for the effect on active migratory movements’ migration speed (> 5 km/h), of NDVI, tailwind, tree cover, season and day/night (excluding transitioning segments between day and night; *N* = 465), with interactions of day/night with the other fixed effects and individual and year as crossed random factors: a) full list of candidate models; b) full model; c) Candidate model (ΔAICc < 4); d) Candidate model (ΔAICc < 4); e) best supported model. In this dataset, migration speeds were higher with increasing tailwind. Migration speeds were still slower over higher NDVI, but conversely, higher during the night compared with the day. Also, the effect of increasing tree cover percentage on migration speed was higher during the day compared to the night. A seasonal effect was also identified here, with the difference in migration speed between day and night being again greater in autumn compared to spring with significantly slower migration speeds at night during spring. **Figure S1.** Migratory routes during (a) autumn 2013 and (b) spring 2014 migration seasons overlaid on to the respective average monthly east – west winds (U wind component) at 850 hPa (c1500 m a.s.l.). Line colors represent the different individuals. East-west wind component is weaker compared to north-south component, facilitating north-south primary movement direction to and from wintering grounds. Data downloaded from ERA5 atmospheric reanalysis product of the European Centre for Medium-Range Weather Forecasts (ECMWF).

## Data Availability

All datasets used and/or analysed during the current study are available from the corresponding author on reasonable request.

## Abbreviations

a.s.l.: Above sea level
AICc: Corrected Akaike’s Information Criterion
ECMWF: European Centre for Medium-Range Weather Forecasts
GLMM: Generalized Linear Mixed Models
GME: Geospatial Modelling Environment
GPS: Global Positioning System
GSM: Global System for Mobile communication
hPa: Hectopascal
LC: Location Class
MODIS: Moderate-resolution Imaging Spectroradiometer
NDVI: Normalized Difference Vegetation Index
PTT: Platform Transmitter Terminals
