# Supplementary material for: Habitat availability influences migration speed, refueling patterns and seasonal flyways of a fly-and-forage migrant
Source: Mov Ecol. 2020 Feb 12;8:10. doi: 10.1186/s40462-020-0190-4 (PMC7017632; doi:10.1186/s40462-020-0190-4)
Supplement: Supplementary file 1 — Additional file 1: Table S1. GLMM results for comparison between actual positions and random points generated within a 50 km buffer zone either side of the flying path, in relation to NDVI, tree cover and elevation for the north part of the migratory routes (latitude 16 N northwards) (N = 901): a) full list of candidate models; b) full model; c) candidate model (ΔAICc <4); d) candidate model (ΔAICc <4); e) candidate model (ΔAICc <4); f) candidate model (ΔAICc <4); g) best supported model. In the drier north (e.g. Sahara desert), the falcons used areas with higher NDVI compared to randomly generated points, avoiding higher elevations. Table S2. GLMM results for comparison between actual positions and random points generated within a 50 km buffer zone either side of the flying path, in relation to NDVI, tree cover and elevation for the south part of the migratory routes (latitude 16 N southwards) (N = 2811): a) full list of candidate models; b) full model, c) candidate model (Δ AICc < 4); d) best supported model. In the more vegetated south they occurred (especially in spring), in areas with higher tree cover, and subsequently higher elevations (particularly in autumn) than what was available to them alongside their routes. Table S3. GLMM results for the effect of all GPS telemetry locations (N = 1435) on migration speed, of NDVI, tailwind, tree cover and season, with interactions of season with the other fixed effects, and individual and year as crossed random factors: a) full list of candidate models; b) full model; c) Candidate model (ΔAICc < 4); d) Candidate model (ΔAICc < 4); e) Candidate model (ΔAICc < 4); f) best supported model. Migration speeds were significantly slower over areas with higher NDVI and tree cover and also significantly slower in spring compared to autumn because of wind effect. Table S4. GLMM results for the effect on migration speed, of NDVI, tailwind, tree cover, season and day/night (excluding transitioning segments between day and night) w [file 40462_2020_190_MOESM1_ESM.docx]

**ADDITIONAL FILE 1**

Full GLMM model results and an additional wind-related figure are presented here to support discussion and conclusions on the main body of the article.

With respect to the routes chosen, in the drier north (e.g. Sahara desert), the falcons used areas with higher NDVI compared to randomly generated points, avoiding higher elevations (Table S1). On the other hand, in the more vegetated south, they occurred (especially in spring) in areas with higher tree cover, and subsequently higher elevations (particularly in autumn) than what was available to them alongside their routes (Table S2).

Migration speeds were significantly slower over areas with higher NDVI and tree cover and also significantly slower in spring compared to autumn because of wind effects (Table S3). When excluding transitioning segments between day and night, slower migration speeds were recorded at night, and over areas with higher NDVI. Differences in migration speed between day and night were greater in autumn compared with spring, with significantly slower migration speeds at night during spring (Table S4). When considering only active migratory movements (migration speed > 5 km/h), migration speeds were higher with increasing tailwind. Migration speeds were still slower over higher NDVI, but conversely, higher during the night compared with the day. Also, the effect of increasing tree cover percentage on migration speed was higher during the day compared to the night. A seasonal effect was also identified here, with the difference in migration speed between day and night being again greater in autumn compared to spring with significantly slower migration speeds at night during spring (Table S5). The east-west wind component was weaker compared to the north-south component, facilitating a north-south primary movement direction to and from wintering grounds. Data were downloaded from the ERA5 atmospheric reanalysis product of the European Centre for Medium-Range Weather Forecasts (ECMWF) [80] (Fig. S1).

Table S1: GLMM results for comparison between actual positions and random points generated within a 50 km buffer either side of the flying path in relation to NDVI, tree cover and elevation (latitude 16N northwards; N = 901): a) full list of candidate models; b) full model; c), d), e) and f) candidate models (ΔAICc < 4); g) best supported model.

| 1. **Full list of candidate models** | | | | | | | |
| --- | --- | --- | --- | --- | --- | --- | --- |
| **s/n** | **Factors** | | | | **AICc** | | **ΔAICc** |
| 1 | (Fix~logtree+logelev+logndvi+logtree:Season+logelev:Season+logndvi:Season+ (1\|Ind)+(1\|Year) | | | | 921.73 | | 5.16 |
| 2 | (Fix~logtree+logelev+logndvi+logndvi:Season+logelev:Season+ (1\|Ind)+(1\|Year) | | | | 919.70 | | 3.13 |
| 3 | (Fix~logtree+logelev+logndvi+logndvi:Season+ (1\|Ind)+(1\|Year) | | | | 919.53 | | 2.96 |
| 4 | (Fix~logndvi+logelev+logndvi:Season+ (1\|Ind)+(1\|Year) | | | | 918.60 | | 2.03 |
| 5 | (Fix~logndvi+logelev+ (1\|Ind)+(1\|Year) | | | | 916.57 | | 0.00 |
| 6 | (Fix~logndvi+logndvi:Season+ (1\|Ind)+(1\|Year) | | | | 936.03 | | 19.46 |
| 7 | (Fix~logndvi+logelev+logelev:Season+ (1\|Ind)+(1\|Year) | | | | 918.41 | | 1.84 |
|  | | **Estimate** | **SE** | **z** | | **P** | |
| 1. **Full model (s/n 1)** | |  |  |  | |  | |
| Intercept | | 7.79 | 2.06 | 3.79 | | < 0.001 | |
| NDVI (log) | | -2.74 | 0.64 | -4.29 | | < 0.001 | |
| Tree (log) | | -0.25 | 0.32 | -0.80 | | 0.42 | |
| Elevation (log) | | 0.63 | 0.23 | 2.72 | | < 0.01 | |
| NDVI (log): Season | | 0.36 | 0.29 | 1.26 | | 0.21 | |
| Tree cover (log): Season | | 0.04 | 0.35 | 0.11 | | 0.91 | |
| Elevation (log): Season | | -0.44 | 0.37 | -1.18 | | 0.24 | |
| 1. **Candidate model (s/n 2)** | |  |  |  | |  | |
| Intercept | | 7.81 | 2.05 | 3.81 | | < 0.001 | |
| NDVI (log) | | -2/74 | 0.63 | -4.34 | | < 0.001 | |
| Tree (log) | | -0.23 | 0.27 | -0.87 | | 0.39 | |
| Elevation (log) | | 0.64 | 0.22 | 2.96 | | < 0.01 | |
| NDVI (log): Season | | 0.37 | 0.29 | 1/30 | | 0.19 | |
| Elevation (log): Season | | -0.46 | 0.34 | -1.37 | | 0.17 | |
| 1. **Candidate model (s/n 3)** | |  |  |  | |  | |
| Intercept | | 7.61 | 2.04 | 3.73 | | < 0.001 | |
| NDVI (log) | | -2.57 | 0.62 | -4.17 | | < 0.001 | |
| Tree (log) | | -0.28 | 0.26 | -1.05 | | 0.29 | |
| Elevation (log) | | 0.48 | 0.18 | 2.67 | | < 0.01 | |
| NDVI (log): Season | | -0.004 | 0.07 | -0.06 | | 0.95 | |
|  | |  |  |  | |  | |
| 1. **Candidate model (s/n 4)** | |  |  |  | |  | |
| Intercept | | 9.23 | 1.36 | 6.81 | | < 0.001 | |
| NDVI (log) | | -3.06 | 0.40 | -7.72 | | < 0.001 | |
| Elevation (log) | | 0.52 | 0.17 | 3.04 | | < 0.01 | |
| NDVI (log): Season | | -0.003 | 0.07 | -0.04 | | 0.97 | |
| 1. **Candidate model (s/n 7)** | |  |  |  | |  | |
| Intercept | | 9.18 | 1.36 | 6.76 | | < 0.001 | |
| NDVI (log) | | -3.05 | 0.39 | -7.73 | | < 0.001 | |
| Elevation (log) | | 0.53 | 0.17 | 3.07 | | < 0.01 | |
| Elevation (log): Season | | -0.04 | 0.09 | 3.07 | | 0.66 | |
| 1. **Best supported model (s/n 5)** | | | | | | | |
| Intercept | | 9.23 | 1.35 | 6.84 | | < 0.001 | |
| NDVI (log) | | -3.07 | 0.39 | -7.80 | | < 0.001 | |
| Elevation (log) | | 0.52 | 0.17 | 3.04 | | < 0.01 | |

Table S2: GLMM results for comparison between actual positions and random points generated within a 50 km buffer either side of the flying path in relation to NDVI, tree cover and elevation (latitude 16N southwards; N = 2811): a) full list of candidate models; b) full model, c) candidate model (Δ AICc < 4); d) best supported model.

| 1. **Full list of candidate models** | | | | | | | |
| --- | --- | --- | --- | --- | --- | --- | --- |
| **s/n** | **Factors** | | | | **AICc** | | **ΔAICc** |
| 1 | (Fix~logtree+logelev+logndvi+logtree:Season+logelev:Season+logndvi:Season+ (1\|Ind)+(1\|Year) | | | | 3355.01 | | 1.28 |
| 2 | (Fix~logtree+logelev+logndvi+logtree:Season+logelev:Season+ (1\|Ind)+(1\|Year) | | | | 3353.73 | | 0.00 |
| 3 | (Fix~logtree+logelev+logndvi+logtree:Season+ (1\|Ind)+(1\|Year) | | | | 3360.04 | | 6.30 |
| 4 | (Fix~logtree+logelev+logtree:Season+ (1\|Ind)+(1\|Year) | | | | 3360.55 | | 6.82 |
| 5 | (Fix~logtree+logelev+ (1\|Ind)+(1\|Year) | | | | 3360.83 | | 7.09 |
| 6 | (Fix~logtree+logtree:Season+ (1\|Ind)+(1\|Year) | | | | 3369.49 | | 15.76 |
| 7 | (Fix~logtree+logelev+logtree:Season+logelev:Season+ (1\|Ind)+(1\|Year) | | | | 3354.57 | | 0.84 |
|  | | **Estimate** | **SE** | **z** | | **P** | |
| 1. **Full model (s/n 1)** | | | | | | | |
| Intercept | | 3.52 | 1.03 | 3.44 | | < 0.001 | |
| NDVI (log) | | -0.53 | 0.28 | -1.85 | | 0.06 | |
| Tree cover (log) | | -0.46 | 0.10 | -4.51 | | <0.001 | |
| Elevation (log) | | -0.27 | 0.14 | -1.94 | | 0.05 | |
| NDVI (log): Season | | 0.18 | 0.22 | 0.85 | | 0.39 | |
| Tree cover (log): Season | | 0.43 | 0.18 | 2.39 | | < 0.05 | |
| Elevation (log): Season | | -0.39 | 0.27 | -1.46 | | 0.14 | |
| 1. **Candidate model (s/n 7)** | | | | | | | |
| Intercept | | 2.03 | 0.53 | 3.82 | | < 0.001 | |
| Tree cover (log) | | -0.56 | 0.09 | -6.53 | | < 0.001 | |
| Elevation (log) | | -0.38 | -0.11 | -3.37 | | < 0.001 | |
| Tree cover (log): Season | | 0.44 | 0.16 | 2.69 | | < 0.01 | |
| Elevation (log): Season | | -0.16 | 0.06 | -2.82 | | < 0.01 | |
| 1. **Best supported model (s/n 2)** | | | | | | | |
| Intercept | | 3.50 | 1.03 | 3.42 | | < 0.001 | |
| NDVI (log) | | -0.46 | 0.27 | -1.68 | | 0.09 | |
| Tree cover (log) | | -0.48 | 0.10 | -4.85 | | <0.001 | |
| Elevation (log) | | -0.34 | 0.12 | -2.92 | | < 0.01 | |
| Tree cover (log): Season | | 0.49 | 0.17 | 2.94 | | < 0.01 | |
| Elevation (log): Season | | -0.17 | 0.06 | -2.88 | | < 0.01 | |

Table S3: GLMM results for the effect on migration speed, of NDVI, tailwind, tree cover and season (all GPS telemetry locations; N = 1435): a) full list of candidate models; b) full model; c), d), and e) candidate models (ΔAICc < 4); f) best supported model.

| 1. **Full list of candidate models** | | | |
| --- | --- | --- | --- |
| **s/n** | **Factors** | **AICc** | **ΔAICc** |
| 1 | (speed~tree*season+NDVI*season+tailwind*season+ (1\|individual)+(1\|Year) | 3895.44 | 5.12 |
| 2 | (speed~tailwind*season+NDVI+ (1\|individual)+(1\|Year) | 3890.42 | 0.10 |
| 3 | (speed~NDVI+season*tree+ (1\|individual)+(1\|Year) | 3893.21 | 2.89 |
| 4 | (speed~NDVI+season+ (1\|individual)+(1\|Year) | 3890.32 | 0.00 |
| 5 | (speed~NDVI+season*tree+tailwind+season+ (1\|individual)+(1\|Year) | 3898.99 | 8.66 |
| 6 | (speed~NDVI+tailwind*season+tree+ (1\|individual)+(1\|Year) | 3891.12 | 0.80 |
| 7 | (speed~tailwind+NDVI+season+tree+ (1\|individual)+(1\|Year) | 3897.84 | 7.52 |

|  | **Estimate** | **SE** | **t** | **P** |
| --- | --- | --- | --- | --- |
| 1. **Full model (s/n 1)** | | | | |
| Intercept | -0.01 | 0.12 | -0.12 | 0.91 |
| Tailwind | 0.04 | 0.03 | 1.05 | 0.30 |
| NDVI | -0.31 | 0.03 | -8.88 | < 0.001 |
| Tree cover | -0.08 | 0.03 | -2.48 | < 0.05 |
| Season | -0.05 | 0.12 | 0.41 | 0.68 |
| Tailwind: season | -0.24 | 0.06 | -3.81 | < 0.001 |
| NDVI: season | 0.06 | 0.08 | 0.67 | 0.51 |
| Tree: season | 0.22 | 0.15 | 1.45 | 0.15 |
| 1. **Candidate model (s/n 3)** | | | | |
| Intercept | -0.02 | 0.11 | -0.15 | 0.89 |
| NDVI | -0.31 | 0.03 | -9.83 | < 0.001 |
| Tree cover | -0.08 | 0.03 | -2.51 | < 0.05 |
| Season | -0.14 | 0.11 | -1.26 | 0.21 |
| Tree: season | 0.22 | 0.13 | 1.70 | 0.09 |
| 1. **Candidate model (s/n 6)** | | | | |
| Intercept | -0.01 | 0.12 | -0.10 | 0.93 |
| Tailwind | 0.04 | 0.03 | 1.03 | 0.30 |
| NDVI | -0.29 | 0.03 | -9.25 | < 0.001 |
| Tree cover | -0.08 | 0.03 | -2.52 | < 0.05 |
| Season | -0.12 | 0.09 | -1.29 | 0.20 |
| Tailwind: season | -0.22 | 0.06 | -3.54 | < 0.001 |
| 1. **Candidate model (s/n 2)** | | | | |
| Intercept | -0.02 | 0.11 | -0.15 | 0.88 |
| Tailwind | 0.04 | 0.03 | 1.05 | 0.30 |
| NDVI | -0.33 | 0.03 | -13.11 | < 0.001 |
| Season | -0.08 | 0.09 | -0.95 | 0.34 |
| Tailwind: season | -0.22 | 0.06 | -3.45 | < 0.001 |
| 1. **Best supported model (s/n 4)** | | | | |
| Intercept | -0.02 | 0.11 | -0.19 | 0.86 |
| NDVI | -0.34 | 0.03 | -13.52 | < 0.001 |
| Season | -0.22 | 0.08 | -2.87 | < 0.01 |

Table S4: GLMM results for the effect on migration speed, of NDVI, tailwind, tree cover, season and day/night (excluding transitioning segments between day and night; N = 984): a) full list of candidate models; b) full model; c) best supported model (Delta AICc < 4).

| 1. **Full list of candidate models** | | | |
| --- | --- | --- | --- |
| **s/n** | **Factors** | **AICc** | **ΔAICc** |
| 1 | (speed~DN*tailwind+DN*NDVI+DN*tree+DN*season+tailwind:season+ (1\|individual)+(1\|Year) | 2585.64 | 25.65 |
| 2 | (speed~DN*NDVI+tree+DN*season+ (1\|individual)+(1\|Year) | 2566.29 | 6.31 |
| 3 | (speed~DN+NDVI+DN:season+tailwind:season+ (1\|individual)+(1\|Year) | 2569.27 | 9.28 |
| 4 | (speed~DN+NDVI+DN:season+ (1\|individual)+(1\|Year) | 2559.99 | 0.00 |
| 5 | (speed~DN*NDVI+DN:season+ (1\|individual)+(1\|Year) | 2564.08 | 4.09 |
| 6 | (speed~DN*NDVI*season+ (1\|individual)+(1\|Year) | 2570.36 | 10.37 |
| 7 | (speed~DN*NDVI+ (1\|individual)+(1\|Year) | 2564.67 | 4.68 |

|  | **Estimate** | **SE** | **t** | **P** |
| --- | --- | --- | --- | --- |
| 1. **Full model (s/n 1)** | | | | |
| Intercept | 0.47 | 0.13 | 3.48 | < 0.05 |
| Day/night | -0.89 | 0.06 | -14.42 | < 0.001 |
| Tailwind | 0.04 | 0.05 | 0.78 | 0.44 |
| NDVI | -0.34 | 0.05 | -7.02 | < 0.001 |
| Tree cover | -0.04 | 0.05 | -0.83 | 0.41 |
| Season | -0.05 | 0.13 | -0.37 | 0.71 |
| Tailwind: day/night | -0.01 | 0.06 | -0.16 | 0.87 |
| NDVI: day/night | 0.12 | 0.07 | 1.75 | 0.08 |
| Tree cover: day/night | -0.08 | 0.07 | -1.04 | 0.30 |
| Season: day/night | -0.34 | 0.18 | -1.93 | 0.05 |
| Tailwind: season | -0.13 | 0.08 | -1.75 | 0.08 |
| 1. **Best supported model (s/n 4)** | | | | |
| Intercept | 0.46 | 0.13 | 3.65 | < 0.05 |
| Day/night | -0.89 | 0.06 | -14.67 | < 0.001 |
| NDVI | -0.33 | 0.03 | -11.10 | < 0.001 |
| Season: day/night | -0.44 | 0.13 | -3.34 | < 0.001 |

Table S5: GLMM results for the effect of NDVI, tailwind, tree cover, season and day/night (active migratory movements’ - migration speed > 5 km/h - excluding transitioning segments between day and night; N = 465): a) full list of candidate models; b) full model; c) and d) candidate models (ΔAICc < 4); e) best supported model.

| 1. **Full list of candidate models** | | | |
| --- | --- | --- | --- |
| **s/n** | **Factors** | **AICc** | **ΔAICc** |
| 1 | speed~DN+NDVI+tree+season+tailwind*DN+DN:season+DN:+DN:tree+DN:season+DN:NDVI+(1\| individual)+(1\|Year) | 1275.05 | 7.58 |
| 2 | speed~DN+NDVI+DN:tree+DN:season+tailwind+(1\|individual)+1\|Year) | 1270.10 | 2.63 |
| 3 | speed~DN+NDVI+DN:tree+DN:season+(1\|individual)+ (1\|Year) | 1267.59 | 0.12 |
| 4 | speed~DN+NDVI+DN:season+tailwind+(1\|individual)+1\|Year) | 1267.47 | 0.00 |

|  | **Estimate** | **SE** | **t** | **P** |
| --- | --- | --- | --- | --- |
| 1. **Full model (s/n 1)** | | | | |
| Intercept | -0.17 | 0.20 | -0.84 | 0.40 |
| Day/night | 0.72 | 0.15 | 4.88 | < 0.001 |
| Tailwind | 0.07 | 0.05 | 1.37 | 0.17 |
| NDVI | -0.23 | 0.06 | -4.13 | < 0.001 |
| Tree cover | -0.04 | 0.06 | -0.62 | 0.54 |
| Season | -0.09 | 0.15 | -0.56 | 0.58 |
| Tailwind: day/night | 0.12 | 0.12 | 0.99 | 0.32 |
| NDVI: day/night | 0.24 | 0.16 | 1.52 | 0.13 |
| Tree cover: day/night | -0.40 | 0.15 | -2.69 | < 0.01 |
| Season: day/night | -1.07 | 0.32 | -3.32 | < 0.001 |
| 1. **Candidate model (s/n 2)** | | | | |
| Intercept | -0.17 | 0.20 | -0.84 | 0.40 |
| Day/night | 0.67 | 0.15 | 4.61 | < 0.001 |
| Tailwind | 0.10 | 0.05 | 2.06 | < 0.05 |
| NDVI | -0.20 | 0.05 | -3.87 | < 0.001 |
| Tree: day/night | -0.30 | 0.10 | -2.86 | < 0.01 |
| Season: day/night | -1.19 | 0.30 | -3.95 | < 0.001 |
| 1. **Candidate model (s/n 3)** | | | | |
| Intercept | -0.16 | 0.19 | -0.86 | 0.39 |
| Day/night | 0.66 | 0.15 | 4.53 | < 0.001 |
| NDVI | -0.19 | 0.05 | -3.66 | < 0.001 |
| Tree: day/night | -0.30 | 0.10 | -2.85 | < 0.01 |
| Season: day/night | -1.17 | 0.30 | -3.89 | < 0.001 |
| 1. **Best supported model (s/n 4)** | | | | |
| Intercept | -0.19 | 0.20 | -0.94 | 0.35 |
| Day/night | 0.70 | 0.15 | 4.81 | < 0.001 |
| Tailwind | 0.10 | 0.05 | 2.06 | < 0.05 |
| NDVI | -0.26 | 0.04 | -5.97 | < 0.001 |
| Season: day/night | -1.13 | 0.30 | -3.73 | < 0.001 |


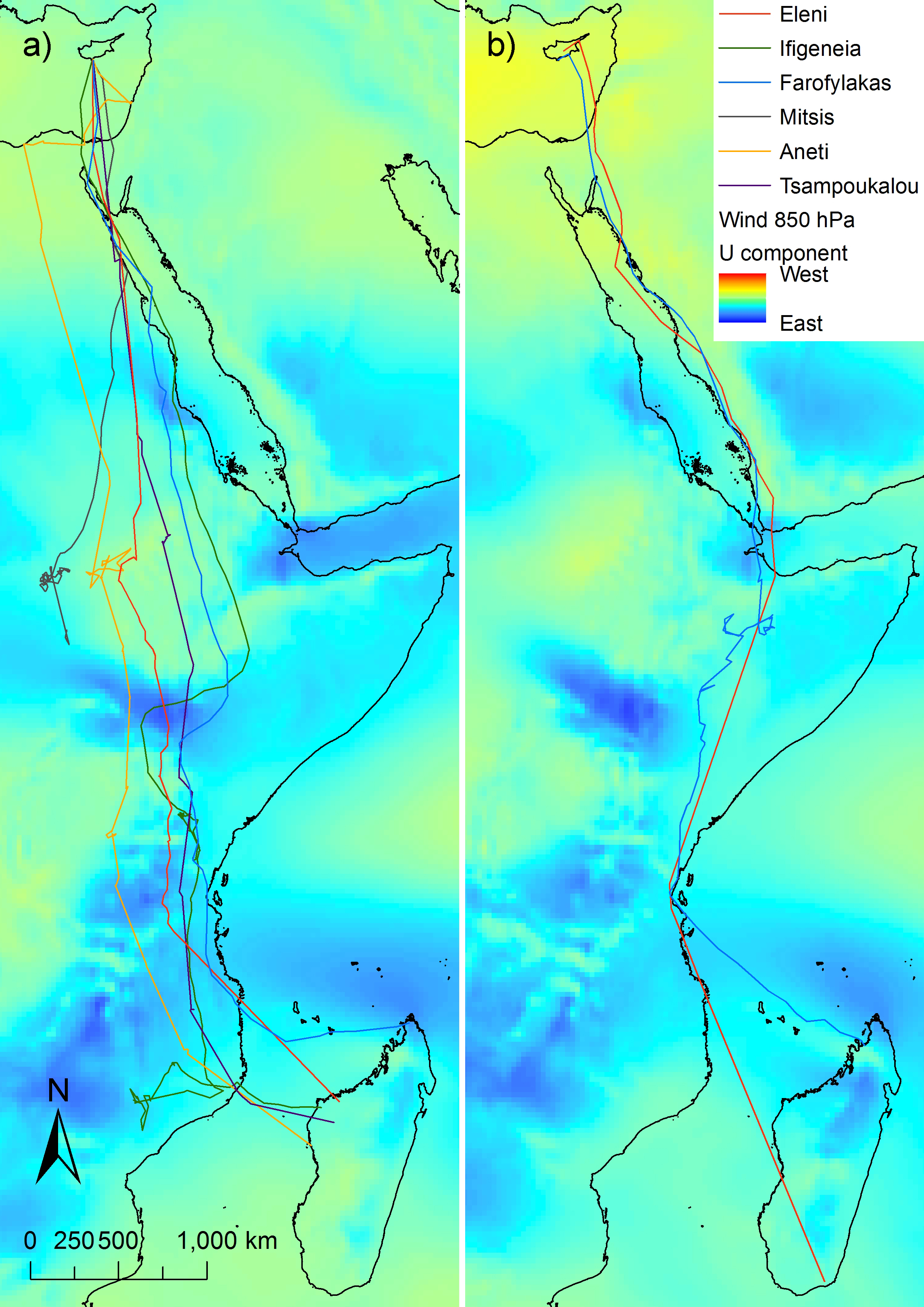


Figure S1: Migratory routes during (a) autumn 2013 and (b) spring 2014 migration seasons overlaid on to the respective average monthly east – west winds (U wind component) at 850 hPa (c1500 m a.s.l.).
